# Evaluation of Epigenetic Age Based on DNA Methylation Analysis of Several CpG Sites in Ukrainian Population

**DOI:** 10.3389/fgene.2021.772298

**Published:** 2022-01-06

**Authors:** N. Kuzub, V. Smialkovska, V. Momot, V. Moseiko, O. Lushchak, A. Koliada

**Affiliations:** ^1^ Institute of High Technologies, Taras Shevchenko National University of Kyiv, Kyiv, Ukraine; ^2^ Institute of Biology and Medicine, Taras Shevchenko National University of Kyiv, Kyiv, Ukraine; ^3^ Diagen Laboratory, Kyiv, Ukraine; ^4^ Precarpathian National University, Ivano-Frankivsk, Ukraine; ^5^ Institute of Food Biotechnology and Genomics NAS of Ukraine, Kyiv, Ukraine

**Keywords:** epigenetics clock, DNA methylation, pyrosequencing, aging, epigenetic age

## Abstract

Epigenetic clocks are the models, which use CpG methylation levels for the age prediction of an organism. Although there were several epigenetic clocks developed there is a demand for development and evaluation of the relatively accurate and sensitive epigenetic clocks that can be used for routine research purposes. In this study, we evaluated two epigenetic clock models based on the 4 CpG sites and 2 CpG sites in the human genome using the pyrosequencing method for their methylation level estimation. The study sample included 153 people from the Ukrainian population with the age from 0 to 101. Both models showed a high correlation with the chronological age in our study sample (R^2^ = 0.85 for the 2 CpG model and R^2^ = 0.92 for the 4 CpG model). We also estimated the accuracy metrics of the age prediction in our study sample. For the age group from 18 to 80 MAD was 5.1 years for the 2 CpG model and 4.1 years for the 4 CpG model. In this regard, we can conclude, that the models evaluated in the study have good age predictive accuracy, and can be used for the epigenetic age evaluation due to the relative simplicity and time-effectiveness.

## Introduction

Aging is significant risk factor for many diseases such as cardiovascular, neurological diseases, diabetes and cancer (Zhao, Stambler, 2020). People, however, do not age at the same rate. There are significant differences in the rate of aging between people, individual differences in their health state, lifespan and predisposition to diseases. Identifying individuals at the greatest risk of accelerated aging, age-related illness, and premature death can be an important opportunity for targeted disease prevention and interventions to prolong both health- and lifespan. The given challenge of biological age estimation is widely solved using quantitative and sensitive molecular biomarkers of diseases and aging.

Among indicators that can predict the risk of age-related diseases and mortality, molecular genetic targets are of the greatest interest. Some of them are the most convenient aging biomarkers, due to the fact that their evaluation does not require a large amount of biomaterial, they reflect key aspects of biological age, and show a connection with diseases (López-Otín et al., 2013). Among a huge variety of molecular markers, epigenetic clocks have recently attracted the greatest interest. Epigenetic clocks use patterns of changes in DNA methylation levels of specific CpG sites in the genome with time for age evaluation. It was repeatedly demonstrated that alterations in methylome are age-associated (Fraga, Esteller, 2007). For instance, DNA methylation levels tend to increase or decrease in some CpGs with age (Ahuja et al., 1998; Issa et al., 2001; Kim et al., 2005; Rakyan et al., 2010; Teschendorff et al., 2010).

The potential medical use of the epigenetic clock is the ability to predict possible morbidity and mortality risks better than chronological age. For example, increased epigenetic age of blood can be a prognosticator of cancer and mortality risks (Levine et al., 2015a; Dugué et al., 2018). Moreover, levels of methylation can shed light on mechanisms of cancer development, which is useful in therapy (Liu et al., 2019). Epigenetic age of cartilage, which was analyzed with Horvath clock, was higher than chronological during osteoarthritis (Vidal-Bralo et al., 2016). Additionally, acceleration of epigenetic age was reported before and during menopause (Levine et al., 2016), Down syndrome (Horvath et al., 2015a), Alzheimer’s disease (Levine et al., 2015b), Werner syndrome (Maierhofer et al., 2017), Huntington’s disease (Horvath et al., 2016) and HIV infection (Horvath, Levine, 2015). Hypomethylation of single CpG site of the fat mass and obesity-associated (FTO) gene in PBMCs can predict type 2 diabetes (Toperoff et al., 2011). Besides, epigenetic age negatively correlates with cognitive and physical abilities (Marioni et al., 2015). Thus, epigenetic clocks can point out fundamental molecular processes, connected with biological age, and serve as powerful tools for analysis of health state, development, and aging.

The epigenetic clock is also shown to be useful in studies of pro-longevity interventions aimed at slowing the rate of aging, such as calorie restriction, supplementation with resveratrol, metformin, and rapamycin together with other approaches for the life- and healthspan extension (Minor, 2011; Wilkinson et al., 2012; Martin-Montalvo et al., 2013; Hubbard, Sinclair, 2014). For instance, a number of studies used epigenetic clocks to test the effect of performed interventions and showed slowed or even reversed epigenetic age in the participants (Fahy et al., 2019; Fitzgerald et al., 2021). A recent study suggests that vitamin D administration also slows down epigenetic age (Chen et al., 2019).

To date actual question is not only to develop credible epigenetic clock models, but also to make them cost and time-effective in order to make the epigenetic age evaluation as a routine procedure. Due to the fact that there are more than 30 epigenetic clocks already developed using different datasets (Oblak et al., 2021), there is a need of validation and examination of the existing clocks on new populations and wide age ranges, rather than developing a new one for the biological age estimation. Such research will help defining the peculiarities of particular clocks usage. In this study, we applied two different epigenetic clock models based on the 4 CpG sites (Bekaert et al., 2015) and 2 CpG sites (Zbieć-Piekarska et al., 2015) for the epigenetic age evaluation of the people from Ukrainian population.

## Materials and Methods

### Samples Processing and Storage

There were 153 people recruited to the study, with 96 women and 57 men aged from 0 to 101 years. We included only people with no family relation in the study. Each study participant signed the informed consent form before enrollment indicating her/his consent to provide a blood sample and to use this sample in the study. Exclusion criteria in the study were: health problems including current chronic diseases, infectious diseases, cancer; refusal to provide informed consent. The study was performed according to the Declaration of Helsinki. The age description of the study sample can be found in Table 1. All whole blood samples were stored at −20°C upon DNA extraction.

**TABLE 1:** Main study sample characteristics.

Age group	Female, n (%)	Male, n (%)	All, n (%)
0–20	13 (13.5)	11 (19.3)	24 (15.7)
21–40	12 (12.5)	12 (21.1)	24 (15.7)
41–60	12 (12.5)	13 (22.8)	25 (16.3)
61–80	14 (14.6)	11 (19.3)	25 (16.3)
81+	45 (46.9)	10 (17.5)	55 (35.9)
**Total**	**96**	**57**	**153**
	**Age (years)**		
Min	0	1	0
Max	101	97	101
Median	78.5	49	63
Mean	65	48.9	59

### DNA Isolation and Bisulfite Conversion

RIBOprep (AmpliSens, Russia) kits were used to extract DNA from the blood samples. Then, DNA concentration was assessed and bisulfite conversion was performed with the EZ DNA Methylation Kit (ZymoResearch, United States) according to manufacturer instructions with the 500 ng of DNA input.

### PCR

For the target sequence amplification, the PCR method was used. PCR was set in the total volume of 25 μl including 10 μl of 2.5x PCR reaction mix (Syntol, Russia), 0.2 pmol of forward and reverse primers, and 40 ng of input DNA. Following conditions were used for the PCR amplification: initial denaturation on the 95°C for 5 min followed by 50 cycles of denaturing on 95°C for 30 s, annealing on 52°C for ASPA gene, 56°C for EDDARAD, 60°C for ELOVL2, 53°C for PDE4C gene for 30 s and extension on 72°C for 30 s followed by the final extension on 72°C for 5 min. Primers for PCR and pyrosequencing were taken from (Daunay et al., 2019) with our modifications (Supplementary materials).

### Pyrosequencing

To assess the DNA methylation level pyrosequencing method was used. To set the pyrosequencing reaction, we used the PyroMark Q24 machine and PyroMark Gold Q24 Reagent kit (Qiagen, Germany) according to the manufacturer’s instructions. For the biotinylated strand capturing streptavidin sepharose beads (GE Healthcare, United States) were used.

### Data Analysis

Obtained data were analyzed with Pyromark Q24 software 2.0.6 version (Qiagen, Germany). Biological age was estimated using formulas described in the (Daunay et al., 2019). For the accuracy estimation coefficient of determination (R^2^), median absolute deviation (MAD), standard error estimation (SEE), and percent of correct predictions (PCP) parameters were used. The determination coefficient reflects the percent of variation explained by the model. It takes a value between 1 and 0, where 1 means that all variation was explained by the model. MAD estimation is the most popular way to evaluate the accuracy of the epigenetic clock models and stands for the mean absolute difference between the predicted and chronological age, thus reflecting the mean error of the epigenetic clock model. SEE exhibits the standard deviation of an estimate and acts as another way of model error evaluation. Percent of correct predictions reflects the percent of the samples with the mean absolute difference between the predicted and chronological age less or same as the chosen diapason of accuracy. Formulas for the accuracy metrics were following:

$$\mathrm{MAD}=\frac{\sum_{i=1}^{n}\left|X_{i}-X_{i}^{'}\right|}{n}$$

$$\mathrm{SEE}=\sqrt{\frac{\sum_{i=1}^{n}{\left(X_{i}-X_{i}^{'}\right)}^{2}}{n-2}}$$

$$\mathrm{PCP}=100*\frac{\sum_{i=1}^{k}\left|X_{i}-X_{i}^{'}\right|}{n}$$

1) X–chronological age
2) X′–predicted age
3) n–number of observations (total)
4) k–a number of observations, which fit in particular error range

For the *p*-value calculation, the Wilcoxon test was used. All the calculations were done with the R software v 4.1.1.

## Results

Initially, we performed linear regression for each of the inspected CpGs in the study for all study sample on the chronological age and estimated the Pearson correlation coefficient and R^2^. All the data obtained, together with the CpG sites description are described in the Supplementary material file. Regression values for single CpGs used further for the epigenetic age evaluation showed high correlations with the chronological age. Among inspected CpG sites, which were included in the models used in the study for the epigenetic age evaluation, the highest Pearson correlation coefficient and R^2^ was obtained for the CpG site in the ELOVL2 promoter region (ELOVL26 position, r = 0.94 and R^2^ = 0.88) and the lowest for the CpG site in the ASPA promoter region (ASPA1 position, r = −0.79 and R^2^ = 0.62).

**FIGURE 1:**
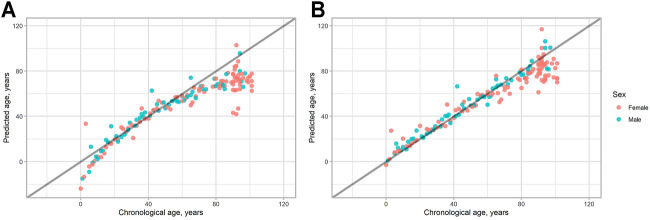
Predicted vs. chronological age for all the study sample. **(A)** 2-CpG model. **(B)** 4-CpG model. The line corresponds to the case when predicted age is the same as chronological age.

In Figure 1 all the data obtained are plotted. As can be seen, people of age above 80 tend to have lower epigenetic age, than their chronological age in both models. Also, in the 2-CpG model young people of age below 18 also tend to have a lower predicted age. To check this, we compared different age groups in both models. The study sample was divided into three different groups regarding their chronological age: young (0–17 years, 21 individuals), adult (18–80 years, 77 individuals), and old (81–101, 55 individuals). Then, the difference between predicted age and chronological age for each person was calculated (delta age = predicted age—chronological age).

**FIGURE 2:**
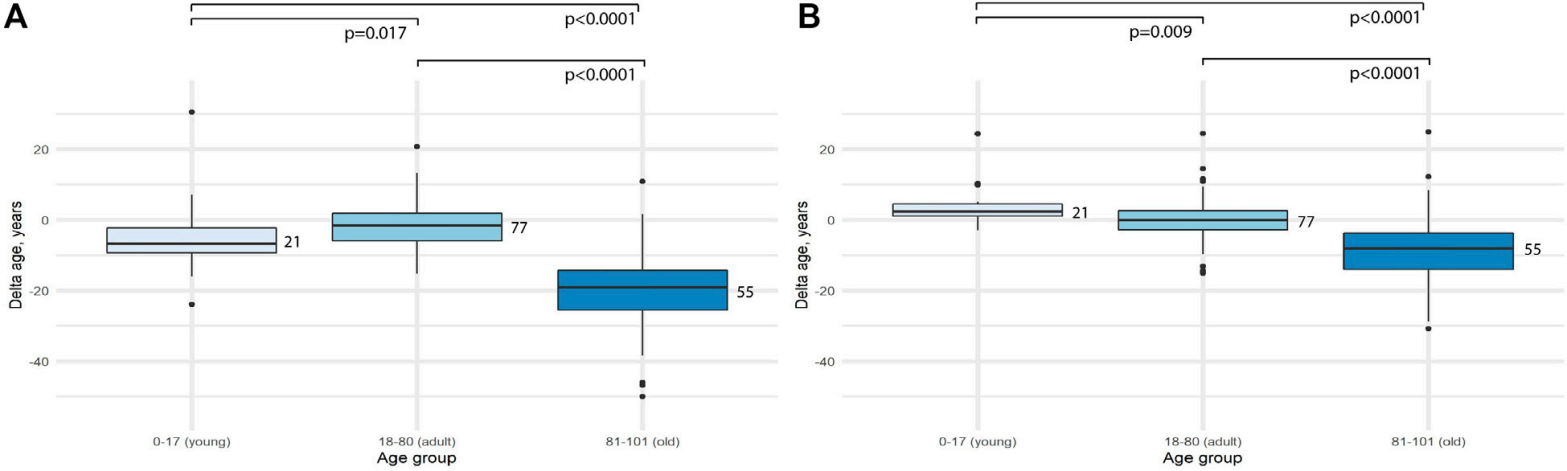
Comparison of the difference between predicted age and chronological age (delta age) for different age groups. **(A)** 2-CpG model. **(B)** 4-CpG model.

Then the difference between delta age for the age groups was assessed. First of all, we compared adult and old groups. It turned out, that the difference was significant for both models (*p* < 0.0001 for 2 CpG-model and *p* < 0.0001 for 4 CpG model). Then, we compared young and adult age groups. The difference was again significant, but the delta age for young people was higher for the 4-CpG model (*p* = 0.009) and lower for the 2-CpG model (*p* = 0.017) (Figure 2).

Taken into account the obtained results we further calculated accuracy metrics separately for each of the groups, and also for all sample studied. R^2^, MAD and SEE are presented in Table 2, PCP (percent correct predictions) can be found in Table 3.

**TABLE 2:** Comparison of the accuracy metrics for different age groups and sexes for 4-CpG model and 2-CpG model.

2-CpG model	Sex	All (0–101)	Young (0–17)	Adult (18–80)	Old (81–101)
R^2^	All	0.85	0.37	0.88	0.02
	Male	0.90	0.73	0.86	0.20
	Female	0.84	0.22	0.91	0.01
MAD, years	All	11.2	9.0	5.1	20.4
	Male	7.4	6.9	5.1	16.4
	Female	13.4	10.9	5.2	21.3
SEE, years	All	15.2	12.2	6.8	23.1
	Male	10.1	9.3	6.8	20.2
	Female	17.6	15.4	6.9	24.2
**4-CpG model**	**Sex**	**All (0–101)**	**Young (0–17)**	**Adult (18–80)**	**Old (81–101)**
R^2^	All	0.92	0.37	0.89	0.03
	Male	0.96	0.73	0.91	0.38
	Female	0.91	0.24	0.89	0.01
MAD, years	All	6.6	4.1	4.1	11.0
	Male	3.9	3.6	3.3	6.6
	Female	8.1	4.5	4.9	12.0
SEE, years	All	9.6	7.0	6.1	13.8
	Male	5.9	5.0	5.6	9.0
	Female	11.2	9.0	6.7	14.8

**TABLE 3:** Comparison of the percent of correct predictions (PCP) for different age groups and sexes for 4-CpG model and 2-CpG model.

2-CpG model	Sex	All (0–101)	Young (0–17)	Adult (18–80)	Old (81–101)
PCP (5-years accuracy)	All	34.0	33.3	55.8	3.6
	Male	47.4	50	56.8	10
	Female	26.0	18.2	55.0	2.2
PCP (7.5-years accuracy)	All	48.4	47.6	77.9	7.3
	Male	64.9	60	81.1	10
	Female	38.5	36.4	75.0	6.7
PCP (10-years accuracy)	All	56.2	76.2	81.8	12.7
	Male	73.7	80	86.5	20
	Female	45.8	72.7	77.5	11.1
**4-CpG model**	**Sex**	**All (0–101)**	**Young (0–17)**	**Adult (18–80)**	**Old (81–101)**
PCP (5-years accuracy)	All	56.9	81.0	74.0	23.6
	Male	75.4	80	81.1	50
	Female	45.8	81.8	67.5	17.8
PCP (7.5-years accuracy)	All	69.9	85.7	85.7	41.8
	Male	86.0	90	89.2	70
	Female	60.4	81.8	82.5	35.6
PCP (10-years accuracy)	All	78.4	90.5	89.6	58.2
	Male	93.0	100	82.5	70
	Female	69.8	72.7	97.3	55.6

**FIGURE 3:**
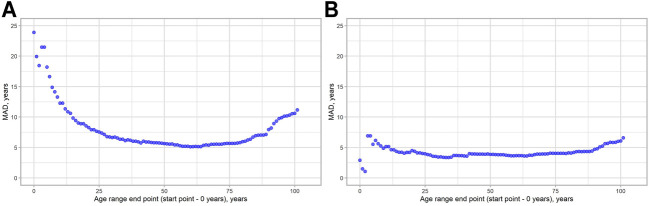
MAD dynamics, depending on the age range chosen. **(A)** 2-CpG model. **(B)** 4-CpG model.

**FIGURE 4:**
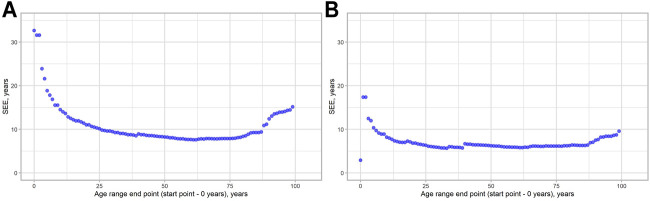
SEE dynamics, depending on the age range chosen. **(A)** 2-CpG model. **(B)** 4-CpG model.

We decided to check, how MAD metrics are changed regarding the age of the people in the study. As can be seen from Figure 3, MAD starts to grow approximately near the age range 0–80 following its maximum in the age range 0–101 for both models. We have done the same evaluation for the SEE to see, whether these two metrics will show similar patterns. The visualization results are shown in Figure 4. Indeed, a similar pattern can be seen here. Of note: higher errors at the beginning of the younger ages are due to smaller age ranges, thus making them more vulnerable to the high error of single data points.

**FIGURE 5:**
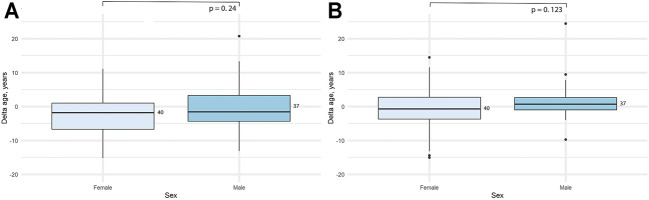
Comparison of the difference between predicted age and chronological age (delta age) for the sexes in the adult (18–80) age group. **(A)** 2-CpG model. **(B)** 4-CpG model.

We also decided to check whether there are any sex-related differences in the data obtained. As previously done for the accuracy metrics, here we also assessed the significance of the difference between delta age both for all the study sample and for each age group separately. Obtained results can be seen in Table 4 and Figure 5. Interestingly, the significant difference in the delta age between the sexes appeared only for the full study sample, while was not observed in separate age groups for the 2-CpG model. In contrast, a significant difference in the all study sample for the 4-CpG model may be explained with the significant difference for the old age group.

**TABLE 4:** Comparison of the significance of the difference between predicted age and chronological age (delta age) for the sexes in the different age groups for the models.

	All (0–101)	Young (0–17)	Adult (18–80)	Old (81–101)
Men	56	10	37	10
Women	97	11	40	45
*p*-value 2 CpG model	**<0.0001**	0.468	0.24	0.23
*p*-value 4 CpG model	**<0.0001**	0.387	0.123	**0.006**

## Discussion

Since the age-dependent changes in DNA methylation were first described in mammals (Vanyushin et al., 1973), and the models based on the CpG methylation levels were used for age prediction (Bocklandt et al., 2011; Koch, Wagner, 2011), many CpG regions have been used in various models for determining epigenetic age (Hannum et al., 2013; Horvath, 2013; Galkin et al., 2021). Despite the fact that most epigenetic clocks use hundreds of CpGs, the predictive power of single epigenetic marks is still high (Daunay et al., 2019). For instance, there is an epigenetic clock based on analysis of only three CpGs which is able to predict age from blood samples with MAD from chronological age of fewer than 5 years (Weidner et al., 2014). Also, an epigenetic clock based on only three loci was developed to estimate the epigenetic age of mice (Han et al., 2018). It has previously been demonstrated that DNA methylation of even single loci can be a reliable indicator of human life expectancy (Zhang et al., 2017). In this study, we attempted to evaluate epigenetic clock models based on several CpG loci using pyrosequencing for the DNA methylation level estimation.

The goal of finding the optimal epigenetic clock is not to develop ideal predictors of chronological age, but to create an easy-to-use tool for testing pro-longevity interventions and tracking changes in the health state of the patients (Han et al., 2018). The Illumina Bead Chip microarray has become the most widely used to assess age-related DNA methylation. However, it was shown previously, that the reproducibility of the data obtained via microarrays can be low (Sugden et al., 2020). On the other hand, determination of DNA for these purposes using deep sequencing technology is still technically difficult and relatively expensive. In addition, not each sequencing cycle covers all the corresponding CpG sites with a sufficient reading depth, and this complicates bioinformatic data analysis and does not allow handling the technique on a daily basis. In this regard, pyrosequencing looks like an alternative solution for epigenetic age estimation, which is more cost and time-effective. Moreover, the pyrosequencing method was shown to be robust for the predefined regions, when it comes to the methylation levels estimation and showed good reproducibility in different laboratories (BLUEPRINT consortium, 2016).

Here we utilized two different epigenetic clocks based on the 2 CpGs and 4 CpGs analysis via pyrosequencing, that were previously described in several papers and tested on different European populations (Bekaert et al., 2015; Zbieć-Piekarska et al., 2015; Daunay et al., 2019). Thus, we can compare the accuracy of the age prediction of given epigenetic clocks in Ukrainian and other populations. We observed the MAD = 5.1 and SEE = 6.8 years for the 2 CpG model and MAD = 4.1 and SEE = 6.1 for the 4 CpG model for the age group of people from 18 to 80 years, which is similar to the previous studies done on the French population (MAD = 6.8, SEE = 8.6 years for the 2 CpG model and MAD = 4.5, SEE = 6.1 for the 4 CpG model), and in the original studies of Bekaert et al., 2015 (MAD = 3.75 for the 4 CpG model) and Zbieć-Piekarska et al., 2015 (MAD = 5.75 for the 2 CpG model). The errors described show that these clocks are suitable for age prediction in different populations, and thus can be used along with other models for age estimation.

When it comes to the comparison of the epigenetic clocks and their accuracy evaluation, we argue that the age group should be considered. As was shown in Figures 3, 4, both MAD and SEE are strongly dependent on the chosen age range in the study. For the epigenetic clock models discussed here, error rates appeared to be the least and relatively stable in the age range between 18 and 80 years. We also observed high variation in the percent of correct predictions between age groups for both models. Due to the inconsistent error distribution, we, therefore, argue that it is much more effective to indicate the accuracy of different epigenetic clocks not only as a mean for the total age range covered in the study but also for the different age ranges. This metric might be important for the epigenetic clock implementation into clinical practice.

We also observed that both delta age (difference between the predicted and chronological age) and errors were significantly higher for the people older than 80, compared to people in the adult age group (18–80 years) in both 4-CpG and 2-CpG models (Figure 2). People in this age group show lower epigenetic age compared to the chronological age, which was also previously shown in another study (Horvath et al., 2015b). These results may be explained in three possible ways.

First, it may be the result of selective mortality of people with higher epigenetic age, thus providing selective advantage to people with younger epigenetic age. The second possible explanation is the inaccuracy of our model. Some currently published epigenetic clocks included results of the DNA methylation of people younger than 80 years in the training set during the model development, or the skewed data with fewer people of old age included (Oblak et al., 2021). Therefore, developed models for the epigenetic clock can be less accurate in age prediction in older age groups. The third explanation might be slowed aging rate in the age of 80 and older, as evidenced by the demographic phenomena of late-life mortality deceleration in human populations (Horiuchi, Wilmoth, 1998; Robine, Vaupel, 2001; Barbi et al., 2018) and in some animal observations (Vaupel et al., 1998). It can be assumed that biological processes that affect the epigenetic clock also contribute to the possible aging deceleration with age. We also measured the difference in delta age (difference between the predicted and chronological age) for both men and women in the age range of 18–80. As can be seen, there was no statistically significant difference observed between the sexes in our study sample. Since our age group of old people (81–101 years) has a distortion in the sex ratio typical of human populations, we did not perform this analysis in the 80 + group. Besides, indicators of epigenetic age in this age group tend to have a significantly higher error rate. Due to this fact, the difference between sexes in the total age range could give the bias, which we wanted to avoid.

## Conclusion

Assessment of epigenetic clocks based on age-dependent changes in methylation of several cytosines in the Ukrainian population showed a high prognostic value in the group of 18–80 years old. The change in epigenetic age, assessed by the described methods for various diseases and preventive interventions, remains unclear. After that, the epigenetic clock based on the pyrosequencing method can be considered as a high throughput, relatively cheap, and reliable tool for estimating the human epigenetic age. However, there is a need to examine the mentioned models to see whether only chronological age can be predicted with these clocks. Further research is required to understand how the epigenetic clock-based indicators of biological age relate to actual aging processes, and how they relate to other well-described indicators for assessing biological age and conditions (Belsky et al., 2018). In addition, further research is needed on the methylation changes estimation in the samples obtained from the same person over different periods of time to determine the accuracy and stability of the different epigenetic clocks. It remains to be seen whether an epigenetic clock based on pyrosequencing, deep sequencing and microarrays can be used to estimate the biological age of individual patients, rather than evaluating epigenetic age in case-control studies for groups of people, which are now widely conducted (Vaquero and Reinberg, 2009; Oblak et al., 2021).

## Data Availability

The data presented in the study are deposited in the Figshare repository, doi:10.6084/m9.figshare.17086406
