# Supplementary material for: Evaluation of Epigenetic Age Based on DNA Methylation Analysis of Several CpG Sites in Ukrainian Population
Source: Front Genet. 2022 Jan 6;12:772298. doi: 10.3389/fgene.2021.772298 (PMC8770732; doi:10.3389/fgene.2021.772298)
Supplement: Supplementary file 1 [file Table1.DOCX]

Supplementary material

Table 1. List of abbreviations and primers used in the study. The highlighted CpG sites abbreviations, correspond to the CpG sites used in 4-CpG and 2 CpG models for the epigenetic age evaluation.

| Gene name | CpG abbreviations | Analysed CpGs positions in the genome (GRCh38) | Primers | Sequence to analyse |
| --- | --- | --- | --- | --- |
| ASPA | **ASPA1** | Chr17: 3,476,273 | Fwd: Biotin-ATTATTTGGTGAAATGATT Rev: CAACCCTATTCTCTAAATCTC Seq: CCCTATTCTCTAAATCTCA | ACRCCATTCTCTAACCAATACTTAACCAAAAATACTCCRATTAACT |
|  | ASPA2 | Chr17: 3,476,237 |  |  |
|  |  |  |  |  |
| EDARADD | **EDARADD1** | Chr1: 236,394,382 | Fwd: TTGGTGATTAGGAGTTTTAGTGTTTT Rev: Biotin-CCACCTACAAATTCCCCAAA Seq: GGAGTTTGTTATGGAAGAAGTAATAGATTG | YGAGAAGATGTTYGTTGG |
|  | EDARADD2 | Chr1: 236,394,370 |  |  |
|  |  |  |  |  |
| ELOVL2 | ELOVL21 | Chr6: 11,044,661 | Fwd: Biotin-AGGGGAGTAGGGTAAGTGAGG Rev: AACAAAACCATTTCCCCCTAATAT Seq: ACAACCAATAAATATTCCTAAAACT | CCRTAAAACRTTGAAGACCRCCRCRCRAAACCRAC |
|  | ELOVL22 | Chr6: 11,044,655 |  |  |
|  | ELOVL23 | Chr6: 11,044,647 |  |  |
|  | ELOVL24 | Chr6: 11,044,644 |  |  |
|  | **ELOVL25** | Chr6: 11,044,642 |  |  |
|  | **ELOVL26** | Chr6: 11,044,640 |  |  |
|  | **ELOVL27** | Chr6: 11,044,634 |  |  |
| PDE4C | **PDE4C1** | Ch19: 18,233,106 | Fwd: AGGTTTGTAGTAGGTTGAG Rev: Biotin-AACTCAAATCCCTCTC Seq: GTTATAGTATGATTAGAGTTT | YGAAGTATTTGTGGYGGTAATTTYGGYGTTTTATTYGTATTTAATAGYGTTTTTATTYGGATTYGGATAAG |
|  | PDE4C2 | Ch19: 18,233,092 |  |  |
|  | PDE4C3 | Ch19: 18,233,083 |  |  |
|  | PDE4C4 | Ch19: 18,233,080 |  |  |
|  | PDE4C5 | Ch19: 18,233,071 |  |  |
|  | PDE4C6 | Ch19: 18,233,059 |  |  |
|  | PDE4C7 | Ch19: 18,233,049 |  |  |
|  | PDE4C8 | Ch19: 18,233,043 |  |  |

Table 2. Pearson Correlation Coeffitients and R^2^ values for the CpGs studied. The highlighted CpG sites, correspond to the CpG sites used in 4-CpG and 2 CpG models for the epigenetic age evaluation.

|  | r | R^2^ | r male | R^2^ male | r female | R^2^ female |
| --- | --- | --- | --- | --- | --- | --- |
| **ASPA1** | **-0.79** | **0.62** | **-0.81** | **0.65** | **-0.77** | **0.59** |
| ASPA2 | -0.81 | 0.65 | -0.87 | 0.75 | -0.77 | 0.59 |
| **EDARADD1** | **-0.91** | **0.83** | **-0.90** | **0.81** | **-0.92** | **0.85** |
| EDARADD2 | -0.79 | 0.63 | -0.85 | 0.73 | -0.77 | 0.59 |
| ELOVL21 | 0.87 | 0.75 | 0.90 | 0.82 | 0.84 | 0.70 |
| ELOVL22 | 0.85 | 0.73 | 0.93 | 0.87 | 0.81 | 0.65 |
| ELOVL23 | 0.91 | 0.83 | 0.92 | 0.84 | 0.91 | 0.82 |
| ELOVL24 | 0.93 | 0.86 | 0.95 | 0.90 | 0.92 | 0.84 |
| **ELOVL25** | **0.90** | **0.80** | **0.93** | **0.87** | **0.88** | **0.77** |
| **ELOVL26** | **0.94** | **0.88** | **0.96** | **0.93** | **0.93** | **0.86** |
| **ELOVL27** | **0.90** | **0.81** | **0.92** | **0.85** | **0.89** | **0.79** |
| **PDE4C1** | **0.85** | **0.73** | **0.94** | **0.89** | **0.81** | **0.65** |
| PDE4C2 | 0.59 | 0.34 | 0.65 | 0.43 | 0.55 | 0.30 |
| PDE4C3 | 0.71 | 0.50 | 0.77 | 0.60 | 0.68 | 0.46 |
| PDE4C4 | 0.58 | 0.33 | 0.75 | 0.56 | 0.50 | 0.25 |
| PDE4C5 | 0.60 | 0.36 | 0.64 | 0.40 | 0.58 | 0.33 |
| PDE4C6 | 0.55 | 0.30 | 0.48 | 0.23 | 0.57 | 0.32 |
| PDE4C7 | 0.53 | 0.28 | 0.43 | 0.19 | 0.59 | 0.35 |
| PDE4C8 | 0.63 | 0.40 | 0.64 | 0.41 | 0.61 | 0.37 |
